# CCR2-positive monocytes contribute to the pathogenesis of early diabetic retinopathy in mice

**DOI:** 10.1007/s00125-022-05860-w

**Published:** 2023-01-26

**Authors:** Aicha Saadane, Alexander A. Veenstra, Martin S. Minns, Jie Tang, Yunpeng Du, Fatima Abubakr Elghazali, Emma M. Lessieur, Eric Pearlman, Timothy S. Kern

**Affiliations:** 1 Department of Ophthalmology, University of California-Irvine, Irvine, CA USA; 2 Case Western Reserve University, Cleveland, OH USA; 3 Institute for Immunology, University of California-Irvine, Irvine, CA USA; 4 Department of Pharmacology, Case Western Reserve University, Cleveland, OH USA; 5 Veterans Administration Medical Center Research Service, Long Beach, CA USA

**Keywords:** CCR2, Diabetic retinopathy, Leucocytes, Leucostasis, Monocytes, Retinal capillary degeneration, Superoxide

## Abstract

**Aims/hypothesis:**

Accumulating evidence suggests that leucocytes play a critical role in diabetes-induced vascular lesions and other abnormalities that characterise the early stages of diabetic retinopathy. However, the role of monocytes has yet to be fully investigated; therefore, we used *Ccr2*^*−/−*^ mice to study the role of CCR2^+^ inflammatory monocytes in the pathogenesis of diabetes-induced degeneration of retinal capillaries.

**Methods:**

Experimental diabetes was induced in wild-type and *Ccr2*^*−/−*^ mice using streptozotocin. After 2 months, superoxide levels, expression of inflammatory genes, leucostasis, leucocyte- and monocyte-mediated cytotoxicity against retinal endothelial cell death, retinal thickness and visual function were evaluated. Retinal capillary degeneration was determined after 8 months of diabetes. Flow cytometry of peripheral blood for differential expression of CCR2 in monocytes was assessed.

**Results:**

In nondiabetic mice, CCR2 was highly expressed on monocytes, and *Ccr2*^*−/−*^ mice lack CCR2^+^ monocytes in the peripheral blood. Diabetes-induced retinal superoxide, expression of proinflammatory genes *Ino*s and *Icam1*, leucostasis and leucocyte-mediated cytotoxicity against retinal endothelial cells were inhibited in diabetic *Ccr2*-deficient mice and in chimeric mice lacking *Ccr2* only from myeloid cells. In order to focus on monocytes, these cells were immuno-isolated after 2 months of diabetes, and they significantly increased monocyte-mediated endothelial cell cytotoxicity ex vivo. Monocytes from *Ccr2*-deficient mice caused significantly less endothelial cell death. The diabetes-induced retinal capillary degeneration was inhibited in *Ccr2*^*−/−*^ mice and in chimeric mice lacking *Ccr2* only from myeloid cells.

**Conclusions/interpretation:**

CCR2^+^ inflammatory monocytes contribute to the pathogenesis of early lesions of diabetic retinopathy.

**Graphical abstract:**

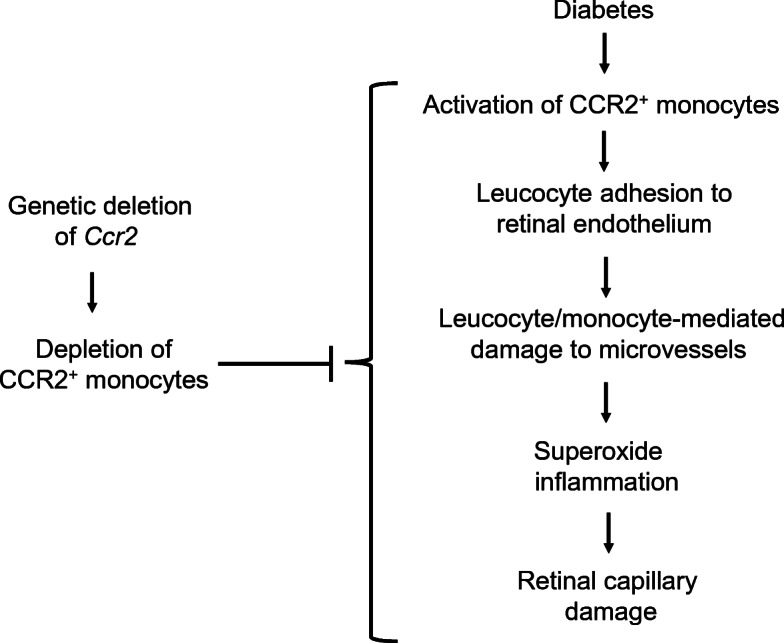

**Supplementary Information:**

The online version contains peer-reviewed but unedited supplementary material available at 10.1007/s00125-022-05860-w.

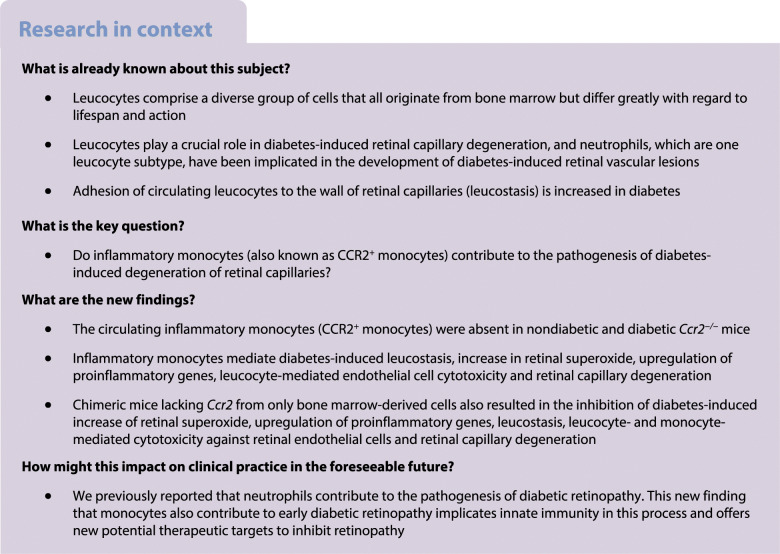



## Introduction

Adhesion of circulating leucocytes to the wall of retinal capillaries (leucostasis) is increased in diabetes. Inhibition of that interaction by deletion of intercellular adhesion molecule 1 (ICAM1) or its ligand, or by expression of neutrophil inhibitory factor (NIF) inhibits diabetes-induced retinal leucostasis, capillary leakage and degeneration of retinal capillaries [1–5]. Selective deletion of proteins involved in oxidative stress and inflammation solely from myeloid-derived cells likewise inhibits the retinal oxidative stress and capillary degeneration [5].

Neutrophils are one leucocyte subtype, and they have been previously implicated in the development of diabetes-induced retinal vascular lesions [6]. Mice with induced diabetes and lacking granulocyte colony-stimulating factor receptor were neutrophil-deficient, and showed significant reduction in diabetes-induced retinal oxidative stress, inflammation, and degeneration of retinal capillaries [5]. Whether or not neutrophils are the only blood cell contributing to the development of retinopathy has not been determined. Monocytes represent another major subset of leucocytes, and they have also been found to play major roles in oxidative stress and inflammation in a variety of diseases [7–10], in part via release of proteases and oxygen-derived free radicals [11].

In mice, like in humans, monocytes represent a heterogeneous group of cells and are commonly divided into classical monocytes (previously called inflammatory monocytes) and nonclassical monocytes (previously called patrolling monocytes) [12–14]. CC chemokine receptor 2 (CCR2) is expressed predominantly by monocytes, and especially by classical inflammatory monocytes. Whole-body deletion of *Ccr2* decreases the abundance of classical monocytes (CCR2^+^) in the blood (a reduction of >84%) [12, 15, 16]. On the other hand, nonclassical monocytes express low levels of CCR2 (CCR2^-^) and high levels of CX3C motif chemokine receptor 1 (CX3CR1) [17]. CCR2 mediates mobilisation of the classical monocyte subset from bone marrow, subsequent migration into sites of injury [15, 18–20], firm adhesion of the cells to the endothelium and extravasation in vivo [21]. The beneficial effect of a CCR2/5 inhibitor on vascular permeability in rodent models of diabetes has been reported previously [22].

The aim of this study was to determine the role of CCR2^+^ inflammatory monocytes in the pathogenesis of diabetes-induced degeneration of retinal capillaries.

## Methods

All procedures involving animals were performed in strict accordance with the National Institutes of Health Guide for the Care and Use of Laboratory Animals, the Association for Research in Vision and Ophthalmology (ARVO) Statement for the Use of Animals in Ophthalmic and Vision Research, and with authorisation of the Institutional Animal and Care Use Committees (IACUC) at Case Western Reserve University, and University of California, Irvine.

### Mice

Wild-type (WT) C57Bl/6J and *Ccr2* knockout (C57Bl/6 background) (*Ccr2*^*−/−*^) mice were obtained from the Jackson Laboratory (Bar Harbor, ME, USA). In all studies, male mice (2–3 months old) were randomly assigned to become diabetic or remain as nondiabetic controls. Diabetes was induced by i.p. injection of a freshly prepared solution of streptozotocin in citrate buffer (pH 4.5) at 60 mg/kg of body weight for 5 consecutive days. Hyperglycaemia was verified at least three times during the second week after streptozotocin administration, and mice having three consecutive measurements of fasting blood glucose >15.3 mmol/l were classified as being diabetic. Insulin was given as needed to prevent weight loss without preventing hyperglycaemia and glucosuria (0–0.2 units of neutral protamine Hagedorn (NPH) insulin s.c 0–3 times per week). All animals were maintained on a standard 12 h light (∼10 lux)−dark cycle and were provided standard rodent chow (Purina TestDiet 5001; TestDiet Richmond, IN, USA) and water ad libitum*.* Blood glucose and HbA_1c_ were measured as reported previously. Body weight was measured weekly. Animals were euthanised and eyes collected at 2 months of diabetes (4–5 months of age) to assess retinal function and biochemistry, and at 8 months of diabetes (10 months of age) to assess retinal capillary degeneration.

Chimeric mice were generated as previously described [5]. Briefly, recipient mice (nondiabetic or diabetic for 2 weeks) were irradiated with two doses of 600 rads, 3 h apart, and subsequently injected intravenously with 3–5 million bone marrow cells from donor mice. Chimeras lacking *Ccr2* from only their marrow-derived cells were generated by transplanting marrow from *Ccr2*^*−/−*^ donors into irradiated WT (C57Bl/6J) hosts (identified as *Ccr2*^*−/−*^→WT). Diabetes was induced prior to irradiation to make sure that irradiation and resulting immune cell damage did not interfere with the induction of diabetes. To control for potential effects of irradiation, nondiabetic and diabetic WT mice were irradiated and transplanted with marrow cells from analogous WT donors (WT→WT).

### Retinal imaging and visual function

We studied retinal structure and function of *Ccr2*^*−/−*^ and WT mice using spectral domain optical coherence tomography (SD-OCT; the 840HHP SD-OCT system, Bioptigen, USA), and electroretinogram (ERG; Diagnosys Celeris rodent ERG device, Diagnosys, USA) recordings [23–25]. Spatial frequency threshold and contrast sensitivity were measured at 2 months of diabetes (5 months of age) with the Virtual Optokinetic system as previously described [26–28]. Briefly, the minimum spatial frequency capable of driving head tracking was determined as the spatial frequency threshold. The experimenter was masked as to the identity of the experimental animals. The contrast sensitivity was measured at spatial frequency of 0.064 cycles/degree.

### Blood phenotype

Mice were euthanised with CO_2_, and blood was collected by cardiac puncture into EDTA-containing tubes (100 mmol/l EDTA). After lysing erythrocytes with RBC lysis buffer (eBiosciences, San Diego, USA) and washing with PBS, antibodies and viability dye were incubated with white blood cells in FACS buffer (PBS with 0.5% BSA and 2 mmol/l EDTA) for 20 min at 4°C. The following antibodies were used (Biolegend, San Diego, USA): CD45-FITC (30-F11 clone), Ly6G-Brilliant Violet 510 (1A8 clone), Ly6C-PE-Cy7 (HK1.4 clone), CD11b-APC (M1/70 clone), F4/80-PE (BM8 clone), CCR2-Brilliant Violet 421 (SA203G11 clone) and CD14-PE-Dazzle 594 (Sa14-2 clone). Viability dye (Fixable Viability Dye eFluor 780, eBiosciences) was added to distinguish live cells. Cells were washed in FACS buffer and fixed for 20 min at 4°C (Perm/Fix buffer, BD Biosciences, USA) before analysis on a Novocyte cytometer (Acea, USA).

### Lucigenin assay of superoxide

Retinal superoxide was measured chemically with lucigenin (bis-*N*-methylacridinium nitrate) [29]. Freshly isolated retinas were pre-incubated in 200 μl of Krebs-Hepes buffer (pH 7.2) with 5 or 30 mmol/l glucose for 10 min at 37°C in 5% CO_2_. Luminescence indicating the presence of superoxide was measured 5 min after addition of lucigenin (5 mmol/l). Luminescence intensity is reported in arbitrary units/mg of protein.

### Quantitative reverse transcription-PCR

Both retinas from each mouse were combined (total of four to six mice per group) and total RNA was isolated with RNeasy Mini kit (Qiagen, USA). Total RNA (0.5 μg) was converted to cDNA by SuperScript III Reverse Transcriptase (Invitrogen from ThermoFisher Scientific, USA) and used for quantitative reverse transcription--PCR (qRT-PCR) conducted on C1000 Touch Thermal Cycler (Bio-Rad, USA). *β-actin* (also known as *Actb*) was used as a housekeeping gene. PCR reactions were performed in triplicate and normalised to *β-actin*.

### Leucostasis

Leucostasis was determined as previously described [30]. Briefly, at 2 months of diabetes, blood was removed from the vasculature of anaesthetised animals by extensive perfusion with PBS via a heart catheter. Subsequently animals were perfused with fluorescein coupled Concanavalin A lectin (20 μg/ml in PBS; Vector Laboratories, USA). Flat-mounted retinas were imaged via fluorescence microscopy and the number of leucocytes adherent to the vascular wall was counted.

### Leucocyte- and monocyte-mediated endothelial cell cytotoxicity ex vivo

Leucocyte-mediated endothelial cell death was determined as previously described [5]. Briefly, mouse retinal endothelial cells (mRECs; Cells Biologics, USA) [31] were grown in DMEM containing 10% FBS and 5.5 or 25 mmol/l glucose. When mRECs were 80% confluent (500,000 cells), leucocytes (100,000; purified from blood with RBC lysis buffer) were added to the mRECs and incubated for 24 h. After 24 h, mRECs were gently rinsed with PBS to remove non-adherent leucocytes, incubated with trypsin for 2 min and washed twice in PBS. The viability of mRECs was measured by trypan blue exclusion with a haemocytometer. Sample identity was masked during counting.

Monocytes were isolated from bone marrow because of the significantly higher yield when using bone marrow compared with peripheral blood (up to 10×10^6^ vs 0.2×10^6^ monocytes per mouse, respectively) [32]. Monocytes were immuno-isolated using EasySep Mouse monocyte isolation kit (StemCell Technologies, USA) following manufacture’s instruction (the purity is up to 94%). Monocyte-mediated endothelial cell cytotoxicity was performed as shown above for leucocytes.

### Diabetes-induced retinal vascular histopathology

After 8 months of diabetes (10 months of age), mice were euthanised and the eyes were then enucleated and fixed in formalin. Retinal vasculature was isolated as previously described [30]. The fixed retina was isolated, rinsed in running water overnight, and then digested with 40 U/ml elastase (Calbiochem, San Diego, USA), 5 mmol/l EDTA, 100 mmol/l sodium phosphate and 150 mmol/l NaCl pH 6.5 at 37°C for 2–3 h. When totally cleaned of neural cells, the isolated vasculature was laid out on a glass microscope slide, dried overnight, stained with haematoxylin and periodic acid−Schiff, dehydrated and mounted with a glass coverslip. Degenerated (acellular) capillaries were quantified in 6–7 field areas corresponding to the mid-retina (×200 magnification) in a masked manner. Acellular capillaries reported per square mm of retinal area were identified as capillary-sized vessel tubes having no nuclei along their length.

### Statistical analysis

Data are expressed as mean ± S.D., except for retinal thickness and ERG measurement that are expressed as mean ± SEM. Statistical analyses were performed with ANOVA followed by Fisher’s test (StatView for Windows software version 5.0.1; SAS Institute, Cary, NC, USA), except for ERG data which was analysed by two-way repeated measures of variance. *p≤0.05; **p≤0.01; and ***p≤0.001.

## Results

### Animals

There was no difference with respect to body weight or blood glucose levels between nondiabetic members of the strains studied. Blood glucose was elevated in all animals assigned to diabetic groups, and the severity of diabetes was not different among the diabetic groups (Table 1).

**Table 1:** Metabolic control in diabetic (2 months of induced diabetes) and nondiabetic WT and *Ccr2*^*−/−*^ mice

Group	*n*	Final body weight (g)	Nonfasting blood glucose (mmol/l)	HbA_1c_ (mmol/mol)	HbA_1c_ (%)
Nondiabetic					
WT	12	34 ± 4	8.5 ± 1.4	10.4 ± 1	3.1 ± 0.1
*Ccr2*^*−/−*^	11	32 ± 2	8.5 ± 1.5	10.4 ± 1	3.1 ± 0.1
Diabetic					
WT	9	27 ± 2	28.0 ± 3.8	70.5 ± 8.7	8.6 ± 0.8
*Ccr2*^*−/−*^	13	29 ± 2	27.8 ± 4.0	68.3 ± 7.6	8.4 ± 0.7

Data are presented as mean ± SD

### The circulating CCR2^+^ monocyte population is absent in nondiabetic and diabetic *Ccr2*^−/−^ mice

**Fig. 1:**
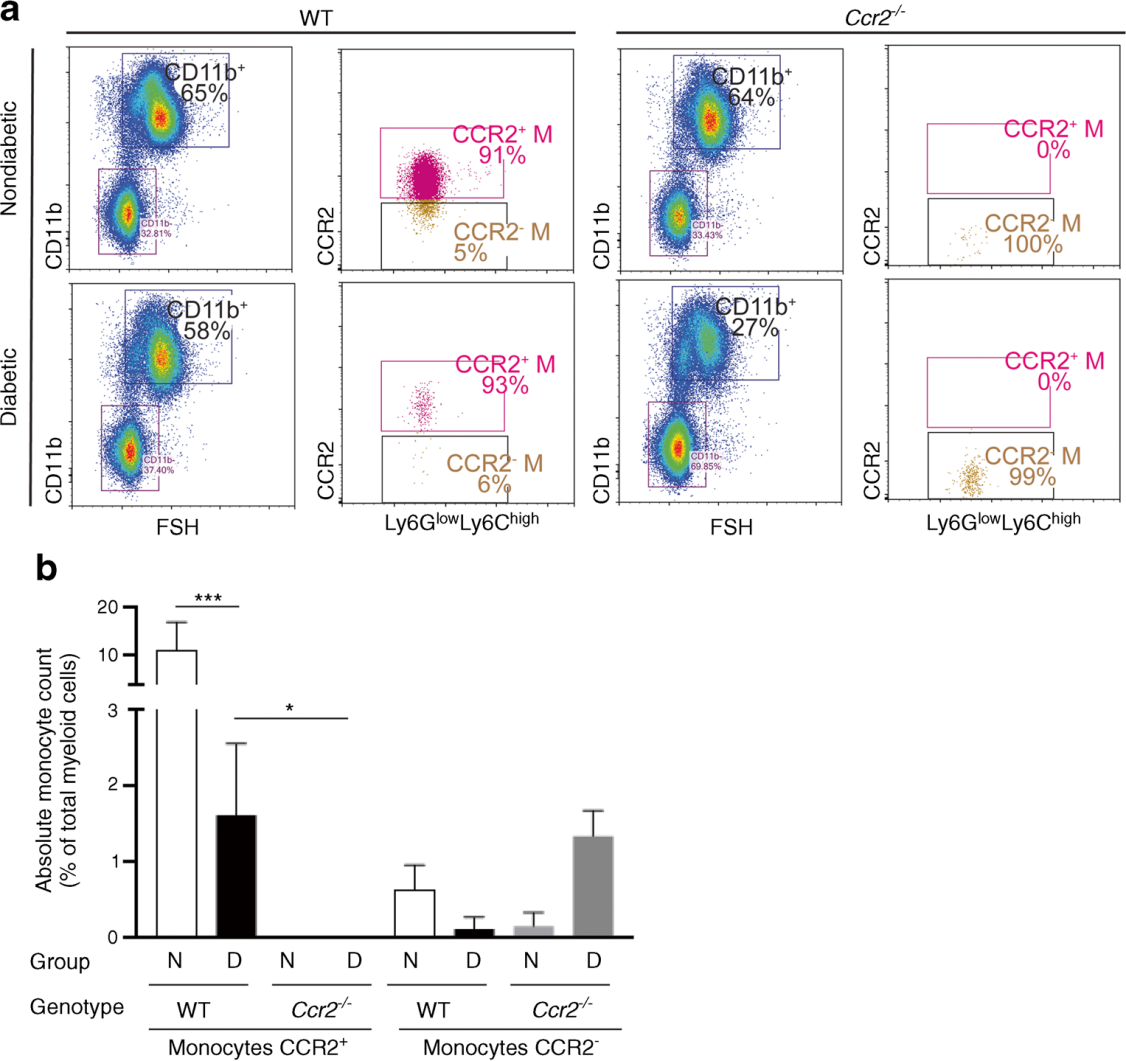
Effect of *Ccr2* deficiency and diabetes on monocytes. Representative flow cytometry dot plots of cell suspensions from the blood of nondiabetic and diabetic WT and *Ccr2*^−/−^ mice (**a**). FSC and SSC identified total leucocytes, CD11b^+^ identified myeloid cells and CD11b, Ly6G and Ly6C staining identified monocytes as CD11b^+^Ly6G^low^Ly6C^high^. CCR2 further identified monocytes as CCR2^high^ or CCR2^low^ monocytes. Numbers in the boxed areas indicate per cent of CD11b^+^, CCR2^high^Ly6G^low^Ly6C^high^ (CCR2^+^ M) or CCR2^low^Ly6G^low^Ly6C^high^ (CCR2^−^ M). Absolute numbers of monocytes are presented as per cent of total number of myeloid cells (**b**). Mean ± SD. *p≤0.05, ***p≤0.001. N, nondiabetic; D, diabetic

It has been reported that *Ccr2* deficiency results in monocyte retention in bone marrow and subsequent depletion from peripheral blood [15]. To determine the effect of *Ccr2* deficiency and diabetes on the subpopulation of monocytes, flow cytometry was performed on whole peripheral blood. Erythrocytes were lysed, and white blood cell suspensions were stained with subset-specific antibodies (CD11b^+^Ly6G^low^Ly6C^high^ for classical monocytes, and CD11b^+^Ly6G^high^Ly6C^high^ for neutrophils). We subsequently used CCR2 expression to differentiate between CCR2^high^ and CCR2^low^ cells. The gating strategy of the flow cytometry data is shown in electronic supplementary material (ESM) Fig. 1. In nondiabetic WT mice, most monocytes were CCR2^high^ (corresponding to classical monocytes). In contrast, this population of cells was completely absent in nondiabetic *Ccr2*^*−/−*^ mice (Fig. 1a,b). In WT mice, the induction of diabetes resulted in the reduction of classical monocytes (Fig. 1a,b), and the majority of neutrophils were CCR2^low^ (ESM Fig. 2). Diabetes did not significantly affect the distribution of neutrophils in both strains of mice (ESM Fig. 2). These results indicate that the circulating leucocytes of *Ccr2*^*−/−*^ mice are deficient in classical monocytes and can therefore be used as a model to study the role of classical monocytes in the development of diabetic retinopathy.

### Neither the absence of CCR2^+^ monocytes nor 2 months of diabetes affects outer nuclear layer thickness

**Fig. 2:**
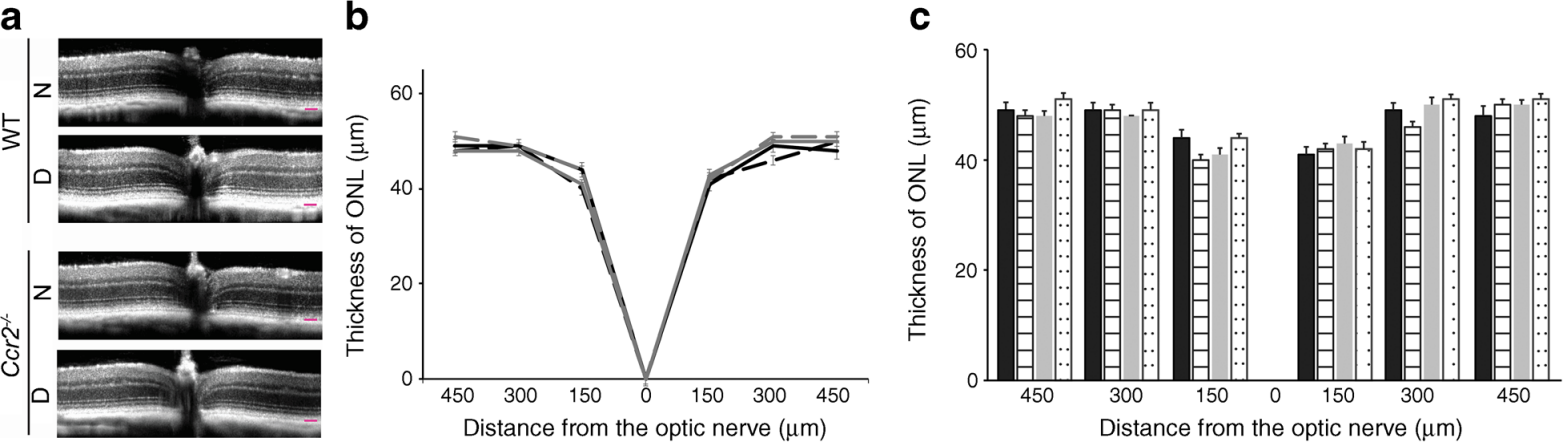
Effect of *Ccr2* deletion and diabetes on outer nuclear layer (ONL) thickness. SD-OCT images (**a**) and quantification of data (spider web and histogram; **b** and **c**, respectively) show that the deletion of *Ccr2* (solid grey lines and grey bars) alone or in conjunction with 2 months of diabetes (grey dashed lines and dotted bars) resulted in no loss of retinal photoreceptors (ONL thickness) compared with nondiabetic (solid black lines and black bars) or diabetic (black dashed lines and striped bars) WT mice. Scale bar, 50 μm. Data are presented as mean ± SD, *n*=8 mice (16 retinas) per group

In vivo examination of mice by high-resolution SD-OCT was used to determine the effect of diabetes and the absence of *Ccr2* on retinal structure and thickness. OCT analysis after 2 months of diabetes (4–5 months of age) indicated that neither diabetes nor the deletion of *Ccr2* caused a significant increase or decrease of outer nuclear layer (ONL) thickness (Fig. 2a,b). We also measured the thickness of total retina along with nerve fibre layer and ganglion cell layer (NFL+GCL) thickness at 300 μm from the optic nerve. The results showed that total retinal thickness (0.24±0.02, 0.23±0.03, 0.23±0.02 and 0.23±0.02 in nondiabetic WT, diabetic WT, nondiabetic *Ccr2*^*−/−*^ and diabetic *Ccr2*^*−/−*^ mice, respectively) and NFL+GCL thickness (0.04±0.01, 0.03±0.01, 0.03±0.01 and 0.03±0.01 in nondiabetic WT, diabetic WT, nondiabetic *Ccr2*^*−/−*^ and diabetic *Ccr2*^*−/−*^ mice, respectively) were not significantly different between all four groups. However, it is worth mentioning that the thickness of inner retina of diabetic animals is controversial; we and others did not detect significant reduction in the inner retina [24, 33–35], whereas others have presented evidence showing significant reduction in the thickness of the inner retina in diabetes [36].

### Inflammatory monocytes mediate diabetes-induced oxidative stress and upregulation of inflammatory genes in the retina

**Fig. 3:**
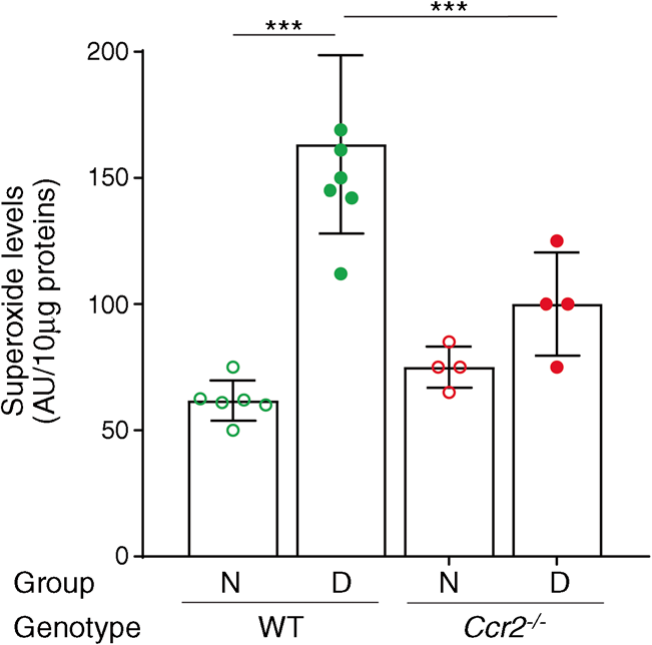
Effect of diabetes and the loss of *Ccr2* on retinal superoxide levels after 2 months of diabetes (4–5 months of age). Superoxide levels in retinas from nondiabetic and diabetic *Ccr2*^−/−^ mice and WT mice are shown. Mean ± SD (*n*=4–8 per group). ***p≤0.001. N, nondiabetic; D, diabetic

**Fig. 4:**
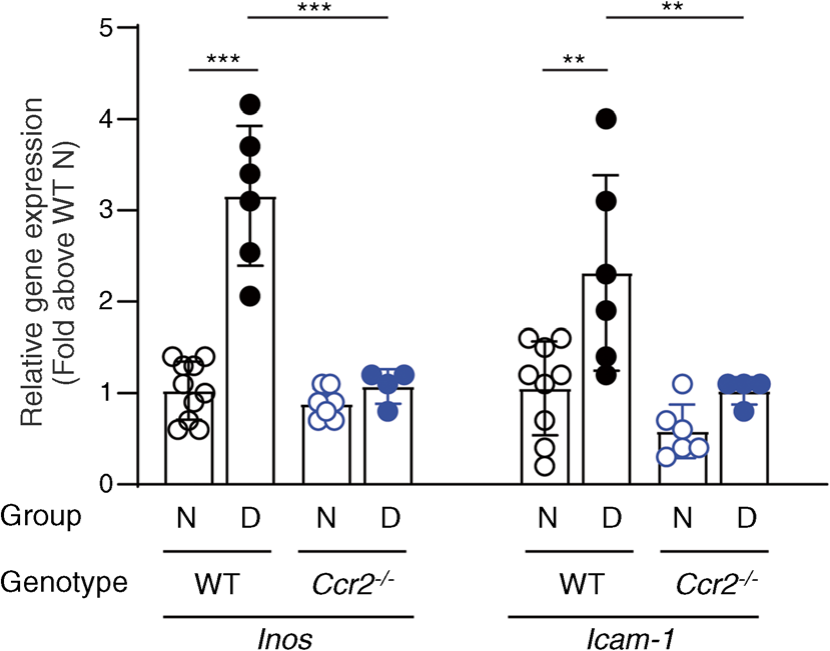
Effect of diabetes and the deletion of *Ccr2* on proinflammatory gene expression in the retina. Genetic deletion of *Ccr2* mitigates diabetes-induced upregulation of *Inos* and *Icam1*. Duration of diabetes was 2 months at the time of this assay. Data are expressed relative to the expression of actin. Data are presented as a per cent of the value of nondiabetic WT controls (*n*=4–10). **p≤0.01; ***p≤0.001. N, nondiabetic; D, diabetic

Oxidative stress has been implicated in the development of diabetic retinopathy [37–42]. To determine if CCR2^+^ monocytes contribute to oxidative stress in diabetic retinopathy, retinal superoxide was measured chemically with the lucigenin method. Compared with nondiabetic WT controls, superoxide levels in the retina were significantly increased in WT mice after 2 months of diabetes. In contrast, retinal levels of superoxide were significantly reduced in diabetic *Ccr2*^*−/−*^ mice compared with diabetic WT mice (Fig. 3). Two months of diabetes also significantly increased the expression of inflammatory genes *Inos* (also known as *Nos2*) and *Icam1* in the retina of WT mice, whereas the diabetic *Ccr2*^*−/−*^ animals showed significantly inhibited expression of both of these proinflammatory genes in the retina compared with diabetic WT mice (Fig. 4).

### CCR2^+^ monocytes mediate a diabetes-induced increase in leucostasis in retinal capillaries

**Fig. 5:**
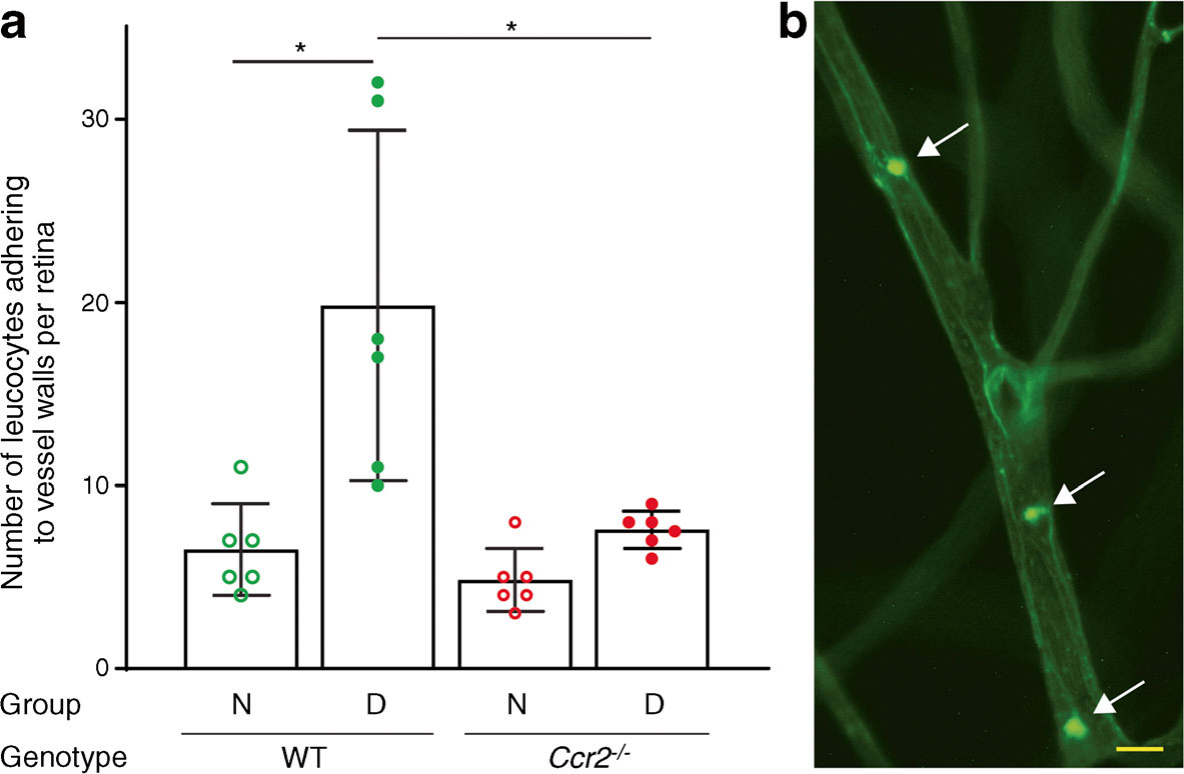
Effect of diabetes and the loss of *Ccr2* on retinal leucostasis. (**a**) Diabetes increases leucostasis in the retina of WT mice, but leucocyte adhesion to retinal capillaries was significantly inhibited in diabetic *Ccr2*^*−/−*^ mice. Leucostasis in retinal microvessels was determined by injection of fluorescein coupled concanavalin A lectin. (**b**) A representative image of leucostasis in the retina of a diabetic mouse (white arrows). Scale bar, 100 μm. Total duration of diabetes was 2 months. Data are presented as mean ± SD, *n*=6 per group. *p≤0.05. N, nondiabetic; D, diabetic

WT mice showed the expected diabetes-induced increase in leucostasis in the retina. In contrast, leucocyte adhesion to retinal capillaries was significantly inhibited in diabetic *Ccr2*-deficient mice (Fig. 5).

### Deficiency of *Ccr2* inhibits leucocyte- and monocyte-mediated cytotoxicity against retinal endothelial cells

**Fig. 6:**
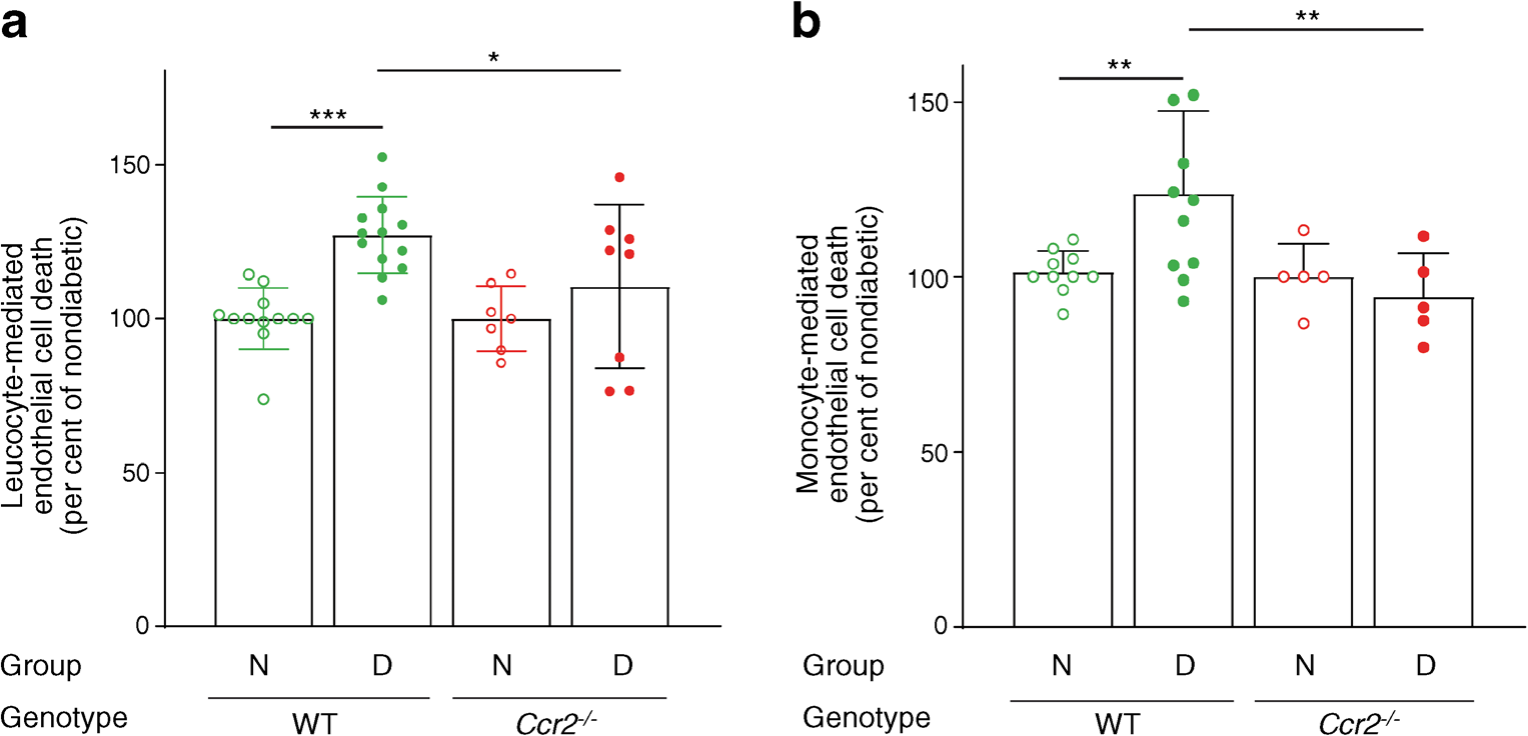
Effect of diabetes and the deletion of *Ccr2* on leucocyte- and monocyte-mediated endothelial cell cytotoxicity. The data are expressed as per cent of corresponding nondiabetic mice. Leucocyte-mediated cytotoxicity towards retinal endothelial cells increased in diabetic WT mice but is significantly inhibited in diabetic *Ccr2*^−/−^ mice (**a**). Similarly, monocyte-mediated cytotoxicity towards retinal endothelial cells was increased in diabetic WT mice and significantly inhibited in diabetic *Ccr2*^−/−^ mice (**b**). Total duration of diabetes was 2 months. Data are presented as mean ± SD, *n*=5–13 per group. *p≤0.05, **p≤0.01, ***p≤0.001. N, nondiabetic; D, diabetic

Ex vivo incubation of leucocytes isolated from WT mice after 2 months of diabetes resulted in more retinal endothelial cell cytotoxicity than leucocytes isolated from nondiabetic WT controls. In contrast, leucocytes harvested from diabetic *Ccr2*^*−/−*^ mice killed significantly fewer endothelial cells when compared with diabetic WT mice (Fig. 6a).

Although data from others [12, 15, 16] and us (Fig. 1) indicates that CCR2 is expressed predominantly on monocytes, we immuno-isolated monocytes to directly evaluate the effect of CCR2^+^ monocytes on endothelial cell cytotoxicity in diabetes. Ex vivo incubation of purified monocytes isolated from WT mice after 2 months of diabetes resulted in significantly more endothelial cell death compared with monocytes harvested from nondiabetic WT controls. In contrast, monocytes isolated from diabetic *Ccr2*^*−/−*^ mice resulted in significantly less endothelial cell death than monocytes harvested from diabetic WT mice (Fig. 6b). This data suggests that CCR2^+^ monocytes (classical monocytes) contribute to endothelial cell death in diabetic retinas.

### Inflammatory monocytes mediate diabetes-induced increase in retinal capillary degeneration

**Fig. 7:**
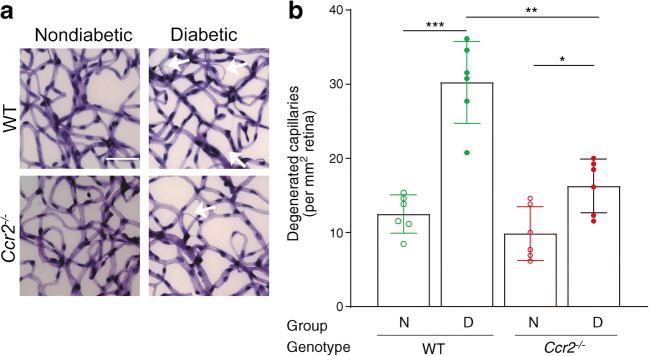
Effects of streptozotocin-induced diabetes on capillary degeneration in retinas from WT and *Ccr2*^−/−^ mice. WT mice diabetic for 32 weeks developed significantly more retinal capillary loss than nondiabetic controls (**a**). *Ccr2* deletion resulted in significantly less retinal capillary loss in diabetes compared with that in diabetic WT mice, but was slightly increased when compared with nondiabetic *Ccr2*^−/−^ mice. Data are graphed as degenerated capillaries per unit area of retina (**b**). White arrows indicate acellular capillaries. Scale bar, 50 μm. Data are presented as mean ± SD, *n*=6 per group. *p≤0.05, **p≤0.01, ***p≤0.001. N, nondiabetic; D, diabetic

Retinal capillary degeneration is one of the most clinically meaningful endpoints of diabetic retinopathy that develop in rodents [24, 43, 44]. As previously reported, diabetes of 8 months in duration significantly increased the number of degenerated capillaries in retinas of WT mice compared with that in nondiabetic WT controls (Fig. 7). In contrast, diabetic mice deficient in *Ccr2* were protected from the retinal capillary degeneration compared with diabetic WT controls (Fig. 7). Since retinal capillary degeneration in diabetic *Ccr2*^*−/−*^ mice was significantly higher than that in nondiabetic *Ccr2*^*−/−*^ mice, however, the data suggest that CCR2^+^ monocytes are not the only determinants of retinal capillary degeneration in diabetes.

### Diabetes-induced increase in retinal superoxide, leucocyte-mediated endothelial cell cytotoxicity and retinal capillary degeneration is mediated by CCR2^+^ monocytes

**Fig. 8:**
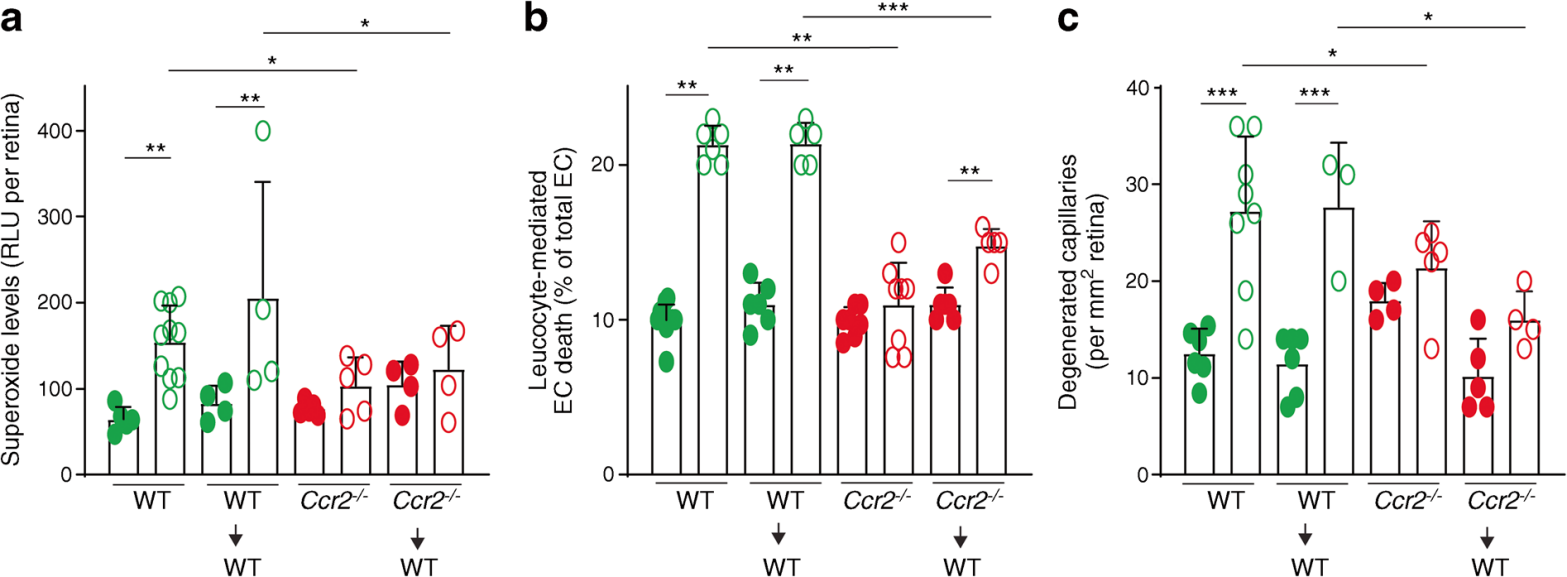
Diabetes-induced retinal superoxide (**a**), leucocyte-mediated endothelial cell (EC) cytotoxicity (**b**) and degeneration of retinal capillaries (**c**) are significantly inhibited in chimeric mice lacking *Ccr2* (*Ccr2*→WT), but not in WT→WT chimeric mice. Total duration of diabetes was 30 weeks. Data are presented as mean ± SD, *n*=4–10 per group. Green symbols indicate WT mice and red symbols indicate *Ccr2*^−/−^ mice. Solid symbols indicate nondiabetic mice and empty symbols indicate diabetic mice. *p≤0.01, **p≤0.01, ***p≤0.001. RLU, relative luminescence units

Although CCR2 is expressed in myeloid cells in nondiabetic animals, it is conceivable that diabetes might result in induction of the receptor elsewhere. To further test the contribution of *Ccr2*-containing myeloid cells in the development of the early stages of diabetic retinopathy, we generated chimeric mice lacking *Ccr2* solely from bone marrow-derived cells. Thirty weeks after the induction of diabetes, diabetic control mice (nonirradiated WT mice and WT→WT mice) showed the expected significant increase in retinal superoxide, leucocyte-mediated killing of endothelial cells, and capillary degeneration (Fig. 8a-c), showing that the irradiation itself did not alter the diabetes-induced pathogenic process that contributes to diabetic retinopathy. In contrast, retinal levels of superoxide, ex vivo killing of retinal endothelial cells by leucocytes, and capillary degeneration were significantly inhibited in diabetic *Ccr2*^*−/−*^ chimeras (*Ccr2*^*−/−*^→ WT) (Fig. 8a-c).

### CCR2^+^ monocytes mediate diabetes-induced reductions of ERG b-wave and spatial frequency threshold, but do not affect contrast sensitivity

**Fig. 9:**
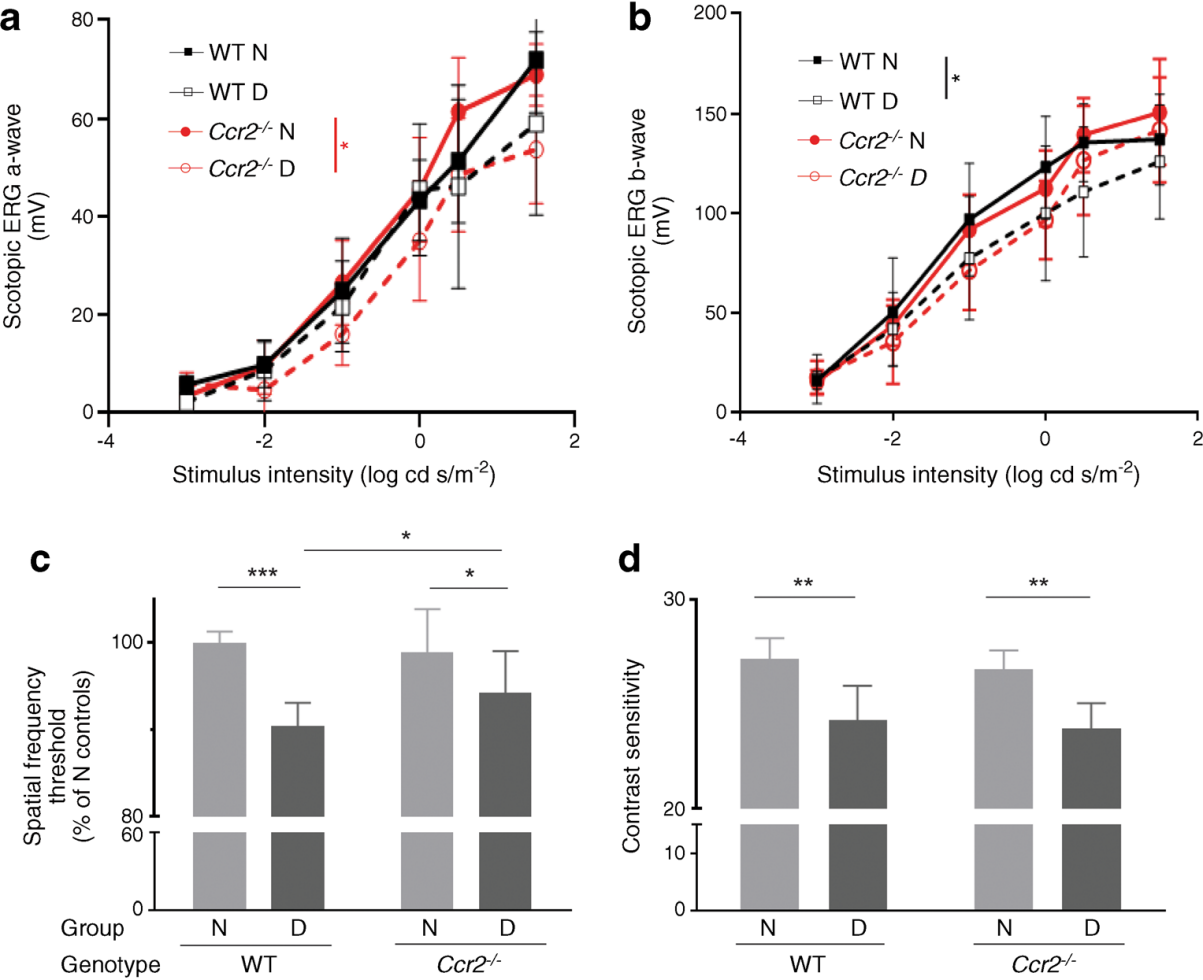
Visual function in nondiabetic and diabetic WT and *Ccr2*^−/−^ mice. (**a**, **b**) ERG a-wave and b-wave amplitudes recorded at 8–10 weeks of diabetes (5 months of age) at increasing light intensities. ERG b-wave amplitudes were significantly reduced in diabetic WT mice, and *Ccr2* deletion further inhibited diabetes-induced b-wave amplitudes in diabetic *Ccr2*^*−/−*^ mice. (**c**) Spatial frequency threshold was significantly reduced in diabetic WT mice, and the lack of *Ccr2* partially inhibited diabetes-induced spatial frequency threshold. (**d**) Contrast sensitivity was impaired in diabetic WT and *Ccr2*^−/−^ mice. Data shown as mean ± SEM (*n*=16–20 eyes). *p≤0.05; ** *p*≤0.01; ***p≤0.001. N, nondiabetic; D, diabetic

Diabetes impairs visual function as assessed by ERG or psychophysical tests, including visual acuity and contrast sensitivity. In this study, we used ERG, spatial frequency threshold and contrast sensitivity to investigate the role of CCR2^+^ monocytes in diabetes-induced visual dysfunction. Consistent with previous studies, 2 months of diabetes significantly decreased ERG b-wave amplitudes (Fig. 9b), spatial frequency threshold (Fig. 9c) and contrast sensitivity (Fig. 9d) in WT mice. *Ccr2* deficiency inhibited the diabetes-induced defects in b-wave amplitude and partially inhibited spatial frequency threshold but had no significant effect on contrast sensitivity (Fig. 9).

## Discussion

Leucocytes play a crucial role in diabetes-induced retinal capillary loss [4, 45, 46]. However, leucocytes comprise a diverse group of cells that originate from bone marrow but differ greatly with regard to life span and action. Determining the leucocyte subtypes involved in the pathogenesis of diabetic retinopathy may help design a novel therapeutic approach to inhibit the retinopathy.

Monocytes, which are a major subpopulation of leucocytes, are part of the innate immune system, where they play a critical role in surveying peripheral tissues and maintaining endothelial cells integrity. However, in some instances, they can contribute to disease development and progression [47–49]. In both humans and mice, monocytes are heterogeneous and have been further divided into several subsets differentially expressing chemokine receptors. CCR2 has been shown to be expressed mainly by classical monocytes (CCR2^high^) [12, 15, 16, 50, 51] (the nonclassical subset does not express CCR2; CCR2^low^), although other leucocyte cells have also been reported to express CCR2 to a lesser extent. The infiltration of inflammatory monocytes was shown to be CCR2-dependent in inflammatory diseases, including several retinal disorders such as retinal injury [8], atrophic age-related macular degeneration [10] and photoreceptor degeneration in models of retinitis pigmentosa [7]. CCR2 is the chemokine receptor for monocyte chemoattractant protein-1 (MCP-1; also known as C-C chemokine ligand 2, CCL2) [50].

It has been shown that the entrance of monocytes into the retina relies on activation of the MCP-1/CCR2 axis [10, 52–54]. In this regard, Sennlaub et al showed that *Ccl2* deficiency (either in *Ccl2*^*−/−*^ mice or as a result of treatment with a pharmacologic inhibitor [RS 102895]) inhibits inflammatory monocyte recruitment to the retina [10]. With regard to diabetic retinopathy, Rangasamy et al showed that the expression of MCP-1 was increased in the retina of diabetic rodents [9], that this expression was accompanied by greater-than-normal numbers of perivascular monocytes in the retina, and that the deletion of *Mcp-1* (also known as *Ccl2*) resulted in significant reduction of monocyte infiltration [9]. Whether or not monocytes contribute to the development of the retinopathy, however, has not been clear. In this study, we focused on the effect of CCR2^+^ monocytes on molecular changes that are characteristic of early stages of diabetic retinopathy (such as oxidative stress and inflammation) and retinal capillary degeneration.

Consistent with previous reports, under steady state condition the majority of peripheral blood monocytes express CCR2 (more than 90%, Fig. 1) [12, 15]. Most neutrophils do not express CCR2 under normal conditions (ESM Fig. 2) [55]. Mice genetically deficient in *Ccr2* had substantially fewer CCR2^+^ monocytes in the peripheral blood [15, 55], however, neutrophil number was not affected. These results suggest that *Ccr2*^*−/−*^ mice could be used as a mouse model to study the role of CCR2^+^ monocytes in the development of diabetic retinopathy.

In the early stages of diabetic retinopathy, an increased number of leucocytes adhere to retinal blood vessels in both diabetic patients and animals [4, 11, 56], potentially contributing to the retinal capillary occlusion that has been observed in diabetes. Consistent with previous reports, we found that diabetic WT mice showed a significant increase in leucostasis in retinal microvessels, and leucocytes from those diabetic WT mice caused more cytotoxicity to retinal endothelial cells. In contrast, leucocytes isolated from diabetic *Ccr2*^*−/−*^ mice and from chimeric mice lacking *Ccr2* only from bone marrow did not cause this leucocyte-mediated endothelial cell death. Since nonclassical monocytes are not significantly affected by *Ccr2* deletion (Fig. 1), we suggest that CCR2^+^ monocytes play an important role in retinal leucostasis and leucocyte-mediated endothelial cell cytotoxicity in diabetic retinopathy.

In order to further investigate if diabetes-induced leucostasis and leucocyte-mediated endothelial cell death could be attributed to CCR2^+^ monocytes, we immuno-isolated monocytes from bone marrow using magnetic bead depletion. Co-culture of mRECS with immuno-isolated monocytes from diabetic WT mice expressing CCR2 resulted in a significant increase in monocyte-mediated endothelial cell cytotoxicity, whereas endothelial cell death was inhibited when cultured with monocytes from diabetic *Ccr2*-deficient mice. These results further demonstrate that CCR2^+^ monocytes play an important role in diabetes-induced leucocyte-mediated endothelial cell cytotoxicity.

Oxidative stress and retinal expression of proinflammatory mediators have been implicated in the development of diabetic retinopathy, and inhibition of retinal oxidative stress in diabetes by antioxidants or overexpression of antioxidant enzymes has been reported to preserve the retinal vasculature in diabetes [41, 57, 58]. Likewise, the inhibition of inflammation has been shown to preserve the retinal vasculature despite hyperglycaemia [46, 59–61], and deletion or inhibition of certain inflammatory proteins or cytokines such as ICAM1, inducible nitric oxide synthase (iNOS) and IL1β inhibited diabetes-induced degeneration of retinal capillaries in diabetic animals [62–64]. Our data showed that retinal superoxide and expression of inflammatory genes were inhibited in *Ccr2*^*−/−*^ mice, suggesting that inflammatory monocytes are implicated in these diabetes-induced abnormalities.

We previously demonstrated a critical role of neutrophils in the development of the early stages of diabetic retinopathy [5, 65]. The finding now that both neutrophils and monocytes contribute to early diabetic retinopathy implicates innate immunity in this disease process. Both neutrophils and monocytes are part of the innate immune system, the first line of defence against toxins, and consist of physical, chemical and cellular defences against various aggressions to tissues. Several reports have shown that both cells are absolutely required for an adequate immune response, and the depletion of one cell population affects the infiltration and/or the function of the other cell [55, 66–70]. For instance, neutrophils attract classical monocytes and facilitate their transmigration, and depletion of neutrophils resulted in decreased classical monocyte infiltration [70]. Likewise, monocytes promote neutrophil accumulation [55]. This new vantage point offers new potential therapeutic targets at which retinopathy might be inhibited.

A CCR2/5 inhibitor has been administered to patients with diabetic macular oedema (DME) [22], but results of that trial indicated that the drug was inferior to monthly ranibizumab (a blood vessel growth inhibitor) with respect to improvement of best corrected visual acuity. The reason for the differing conclusions from this patient study and our pre-clinical study are not clear, but it is possible that the duration of drug administration to patients was too short to demonstrate an effect (only 12 weeks). In addition, it seems likely that the degree of CCR2 inhibition played an important role; *Ccr2* expression in our *Ccr2*^*−/−*^ mice was totally inhibited, whereas the drug administered to patients only blocked the receptor by an unknown amount. Perhaps CCR2 inhibition has a stronger effect on capillary degeneration (studied in the present report) than it has on capillary permeability (studied in the clinical report). Additional studies will be required to determine if more potent inhibitors of CCR2 offer meaningful clinical benefit to patients with diabetes.

Our major findings showed that the whole-body deletion of *Ccr2* (*Ccr2*^*−/−*^ mice) and chimeric mice lacking *Ccr2* only from bone marrow-derived cells in mice resulted in the inhibition of diabetes-induced increases of retinal superoxide, upregulation of proinflammatory genes (*Inos* and *Icam1*), leucostasis, leucocyte- and monocyte-mediated cytotoxicity against retinal endothelial cells, and most importantly, retinal capillary degeneration. The absence of inflammatory monocytes also mitigated diabetes-induced visual dysfunction, notably in ERG b-wave. These results demonstrate that monocytes (and innate immunity) contribute to at least the vascular lesions of early diabetic retinopathy.

## Supplementary information

ESM (PDF 938 kb)

## Data Availability

The data generated and presented in the present study are available from the corresponding author on reasonable request.

## Abbreviations

CCR2: CC chemokine receptor 2
ERG: Electroretinogram
GCL: Ganglion cell layer
ICAM1: Intercellular adhesion molecule 1
mREC: Mouse retinal endothelial cell
NFL: Nerve fibre layer
SD-OCT: Spectral domain optical coherence tomography
WT: Wild-type
