# Supplementary material for: CCR2-positive monocytes contribute to the pathogenesis of early diabetic retinopathy in mice
Source: Diabetologia. 2023 Jan 26;66(3):590–602. doi: 10.1007/s00125-022-05860-w (PMC9892100; doi:10.1007/s00125-022-05860-w)
Supplement: Supplementary file 1 — (PDF 938 kb) [file 125_2022_5860_MOESM1_ESM.pdf]

Title:

CCR2-positive monocytes contribute to the pathogenesis of early diabetic retinopathy in mice

Aicha Saadane[1], Alexander A. Veenstra[2], Martin S. Minns[3], Jie Tang[4], Yunpeng Du[1], Fatima Abubakr Elghazali[1], Emma M. Lessieur[1], Eric Pearlman[3], Timothy S. Kern[1,5]

[1]Department of Ophthalmology, University of California-Irvine, Irvine, CA, USA
[2]Case Western Reserve University, Cleveland, OH, USA
[3]Institute for Immunology, University of California-Irvine, Irvine, CA, USA
[4]Department of Pharmacology, Case Western Reserve University, Cleveland, OH, USA
[5]Veterans Administration Medical Center Research Service, Long Beach, CA, USA

ORCID ID:
Aicha Saadane **0000-0001-9985-2147**

Correspondence to: Aicha Saadane, asaadane@hs.uci.edu

Received: 17 May 2022 / Accepted: 13 September 2022

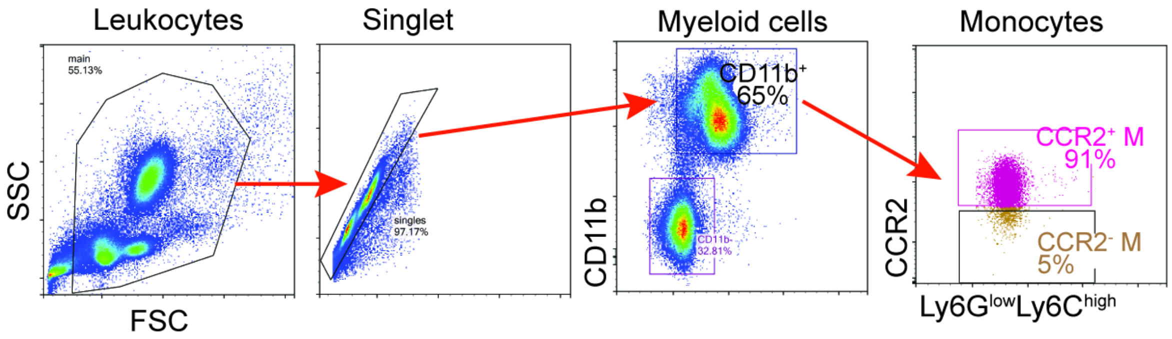

ESM Fig.1 Gating stategy of the flow cytometry for identifying monocyte subsets. FSC and SSC identified total leukocytes, $CD11b^{+}$ identified myeloid cells and CD11b, Ly6G and Ly6C staining identified monocytes as $CD11b^{+}Ly6G^{low}Ly6C^{high}$ and CCR2 further identified monocytes as $CCR2^{+}$ or $CCR2^{-}$. SSC, side scatter; FSC, forward scatter.

a

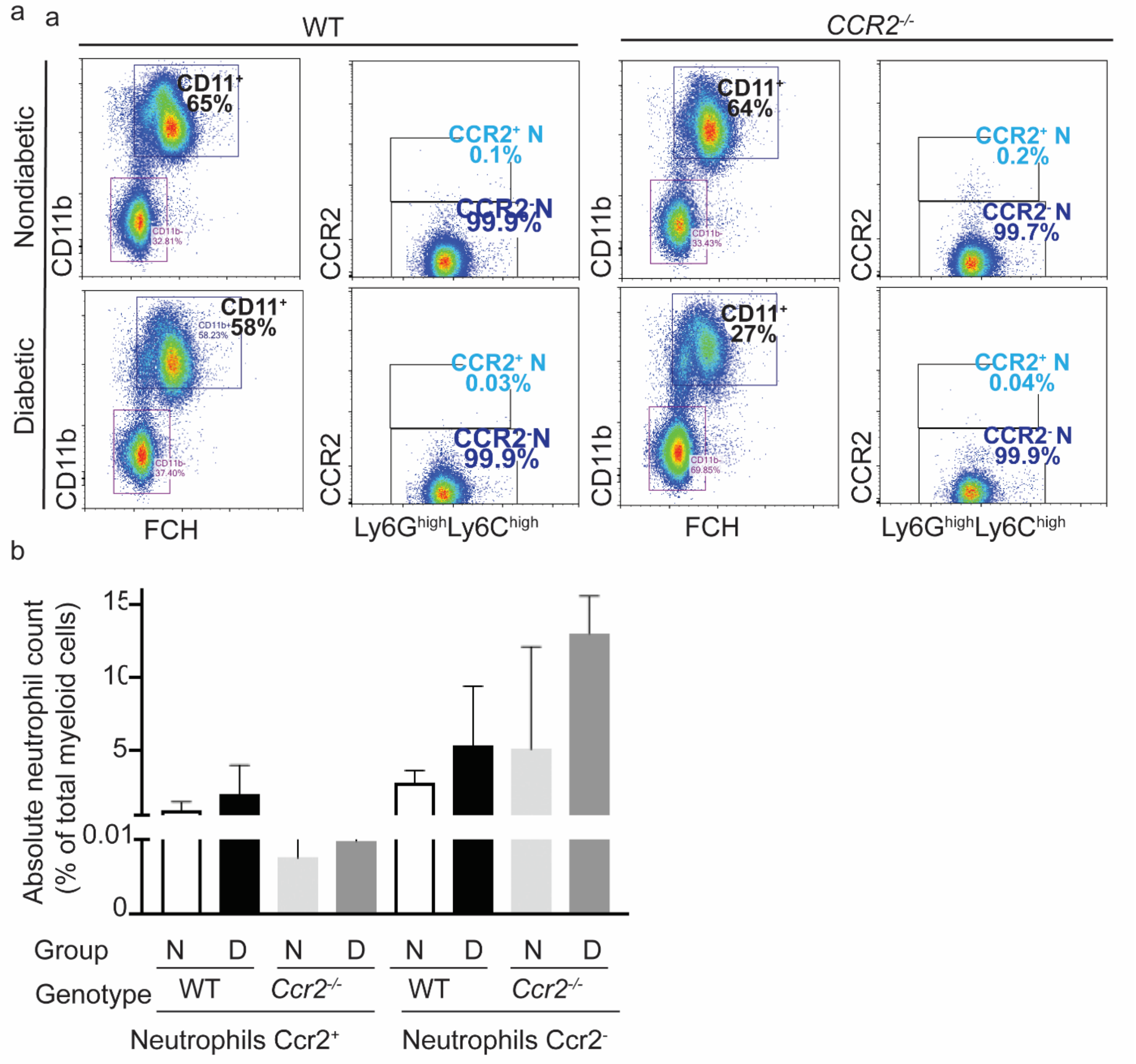

Supplementary Figure 2. Effect of *Ccr2* deficiency and diabetes on Neutrophils distribution. FSC and SSC identified total leukocytes, CD11b^{+} identified myeloid cells and CD11b, Ly6Ghi and Ly6C staining identified neutrophils as CD11b^{+}Ly6G^{high}Ly6C^{high} and CCR2 further identified neutrophils as CCR2^{+} or CCR2^{-}. Flow cytomety of the blood of nondiabetic and diabetic mice of WT and *Ccr2*$^{-/-}$ mice (a). Absolute numbers of monocytes were normalized to the total number of myeloid cells (b). Mean ± SD. N, nondiabetic; D, diabetic.
